# An engineering biology approach to automated workflow and biodesign

**DOI:** 10.1093/synbio/ysae009

**Published:** 2024-06-15

**Authors:** Alexis Casas, Matthieu Bultelle, Richard Kitney

**Affiliations:** Department of Bioengineering, Imperial College London, London, Westminster SW7 2BX, UK; Department of Bioengineering, Imperial College London, London, Westminster SW7 2BX, UK; Department of Bioengineering, Imperial College London, London, Westminster SW7 2BX, UK

**Keywords:** biodesign, DBTL paradigm, DAGs, laboratory automation

## Abstract

The paper addresses the application of engineering biology strategies and techniques to the automation of laboratory workflow—primarily in the context of biofoundries and biodesign applications based on the Design, Build, Test and Learn paradigm. The trend toward greater automation comes with its own set of challenges. On the one hand, automation is associated with higher throughput and higher replicability. On the other hand, the implementation of an automated workflow requires an instruction set that is far more extensive than that required for a manual workflow. Automated tasks must also be conducted in the order specified in the workflow, with the right logic, utilizing suitable biofoundry resources, and at scale—while simultaneously collecting measurements and associated data. The paper describes an approach to an automated workflow that is being trialed at the London Biofoundry at SynbiCITE. The solution represents workflows with directed graphs, uses orchestrators for their execution, and relies on existing standards. The approach is highly flexible and applies to not only workflow automation in single locations but also distributed workflows (e.g. for biomanufacturing). The final section presents an overview of the implementation—using the simple example of an assay based on a dilution, measurement, and data analysis workflow.

## Graphical Abstract

**Figure:**
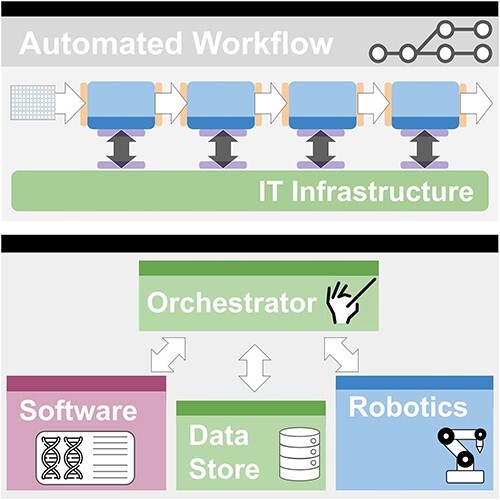

## 1. Introduction

Engineering biology has been identified by both the US and UK governments as a key technology for the global transition to a bio-based economy (e.g. The White House Executive Order on Advancing Biotechnology and Biomanufacturing Innovation—2022 (1)). This applies to the replacement of fossil-based products with renewable biological resources to produce, for example, chemicals, consumer and industrial goods, energy, and food (2). Applications of engineering biology often need to run operations on biological samples at very large scales. This is often due to the need to to explore vast design spaces of genetic diversity. Another issue is the need for ever greater reliability and replicability. This is of great importance in industrial settings and is a rising concern in biological research. Several studies have shown that results produced in leading journals by one group are frequently impossible to replicate by other groups (3). Reasons for poor replicability range from human error to different methodologies, to insufficient control of experimental factors, not to mention underpowered studies.

To address these problems, there is a need to introduce more automation—and to exploit its capacity for higher throughput and higher replicability. Automation in biology is not new. For example, chemostats were invented in the 1950s (4), and the introduction of liquid-handling robots in the 1990s was instrumental in the development of high-throughput screening (5). The push for automation has recently accelerated due to the establishment of biofoundries (6). Biofoundries are specialized laboratories that combine software-based design and automated pipelines to build and test genetic devices (7). To enable rapid design, construction and testing, biofoundries are organized around the Design–Build–Test–Learn (DBTL) cycle (6). They are essential components of a wider ecosystem of facilities supporting the bioeconomy—specifically tasked with application development, scale-up and commercialization (8). They are also ideal test beds for the integration of automated platforms and dedicated software tools.

## 2. Workflow automation

In this paper, we will address a problem that rapidly becomes apparent when working in a biofoundry: the problem of workflow automation.

**Figure 1.**
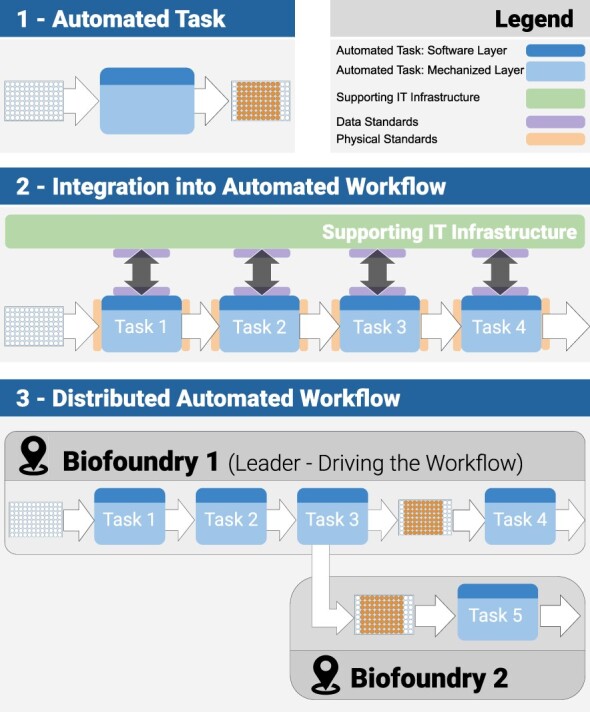
Automated tasks, automated workflows and distributed automated workflows.

In the hands of a skilled human operator, implementing a standard laboratory-based workflow can be relatively straightforward (often requiring a minimum set of instructions and relying on personal expertise). Automation of a workflow in a biofoundry is more complex—necessitating the integration of separate automated tasks (Figure 1). This process, and the associated architecture, will now be described in more detail. Referring to Figure 1, the top of the diagram (Figure 1—section 1) describes an automated task. Each automated task has two components—a software layer coupled to a mechanized layer. Instructions for the task are communicated to the mechanized layer by the software layer, so that the automated platform (e.g. a liquid-handling robot) accomplishes the specified task.

The second area of Figure 1 (section 2) illustrates how multiple tasks are combined into an integrated automated workflow. In the figure, a generic workflow is described, comprising four tasks—performed sequentially on one or more microplates (e.g. creation, culture, and some measurement, etc.). The instructions for any task in the workflow are transmitted to the software layer of the task and then to its mechanized layer (as previously described). In addition, there is a supporting IT infrastructure that is in charge of sending the instructions (in the order of the workflow) and collecting all the generated data (including the operational and experimental data) before curating, organizing and storing them—while monitoring the advancement of the workflow.

To be able to create a sequence of automated tasks in such a manner requires more than a dedicated supporting IT infrastructure. A significant degree of standardization (and, therefore, standards) is also needed. Technical standards are a crucial enabler of integration efforts. They provide the interface between different processes—reducing incompatibility and removing potential sources of error (thus improving reliability and replicability). Several data standards already exist in engineering biology—together with more general standards in communication systems—these standards are indicated in Figure 1. Similarly, physical standards are needed for the equipment involved in the workflow—for instance, the ANSI standards for microplates (9). More work is needed however, especially regarding details of data and metadata (10).

Once automated protocols have been developed and tested, little human involvement is required—reducing human-induced errors and variability (a core promise of automation). Porting protocols between biofoundries (running different equipment, often controlled by different codes) is less straightforward. However, recent approaches, such as programmatically open platforms like Opentrons (11), or platform-agnostic languages, such as LabOP (12) and PyLabRobot (13), are very promising technologies in this regard. They also begin to address a distributed future, where once the workflow has been installed in an individual biofoundry, it can be implemented in multiple biofoundries, often with relatively minor modifications. Elements of any automated workflow can then be distributed to other locations (possibly with significant geographical separation)—provided such locations use compatible infrastructures and carry out the necessary protocol adjustments. Figure 1, section 3 shows an example where a ‘sacrificial plate’ is created for specific measurements to be conducted in a different location with special expertise in this aspect of the workflow. For example, for a DBTL strategy, specifications for the design may be undertaken in the USA, the design in Sweden, modeling in Singapore, the build in China and testing and debugging again performed in the USA. It is also possible to envisage distributed manufacturing based on a similar set of concepts—for instance, the distributed manufacture of vaccines and their hundreds of individual components at multiple local sites (14).

## 3. Implementation

The next sections describe a general and flexible approach to the development of automated workflow. This combines directed acyclic graphs (DAGs) and orchestrators, high levels of automation and a distributed information system. The discussion that follows is in the context of biodesign—but the principles presented extend to other applications. The methodology presented is intended to be implemented in a biofoundry—but any location with a sufficient degree of automation will derive benefit.

Biodesign is a multistage iterative process (its workflow underpinned by the DBTL paradigm (15)). The design stage is undertaken in the dry lab and the build and test stages in the wet lab. In engineering biology terms, this means that the design is undertaken using a suite of software (fully amenable to automation, as it is purely computational), while the build and test stages are conducted in a biofoundry.

Computer-based biodesign has been typically based on a design abstraction hierarchy mirroring electronic circuit design. Implementation requires an infrastructure with standard, well-characterized biological components used in the design. This, in turn, requires standardized data formats and analysis procedures, standardized data repositories and a range of design tools that are capable of integration. Of particular importance in this strategic approach is a set of standard components drawn from registries (16). These are incorporated into the design—with the subsequent steps of computer modeling, characterization, testing and validation. At the simplest level, components may only comprise sequence information—e.g. in an SBOL file (17). At the other end of the spectrum, components may be organized in terms of type and function—with full characterization data, metadata and access to the raw data from characterization experiments (18). Recently, artificial intelligence approaches (19) have been proposed to address all the contextual effects affecting biodesign and preventing direct use of characterization data as originally intended. More generally, biodesign still lacks a user-friendly framework for design and troubleshooting (20). Without validated models with good prediction, many assays are needed to test and validate the designs. This necessitates effective automation of the build and test stages in a biofoundry, and more generally, the processing of biological samples in engineering biology.

Implicit in the automated implementation of a workflow is the requirement to translate the high-level (human-readable) description of a workflow into low-level, machine-readable instructions, so that automated biofoundry resources (hardware or software) can carry out the separate operations in the workflow—while also ensuring these operations are conducted in the right order and with the right logic.

Our proposed solution is based on a three-tier hierarchical model. At the top level is the human-readable workflow; at the second level, the procedures for data and machine interaction; and at the third level, the automated implementation in the biofoundry.

The second level is the key stage of this hierarchy. We use DAGs for the representation of workflows, and orchestrators for their execution. A DAG is a directed graph that, in the context of a workflow, comprises arcs connecting steps in the workflow sequentially, making it acyclic (i.e. with no loops). An orchestrator is a type of software very common in data processing applications (21). Its purpose is to execute workflows, assign tasks to resources, and schedule, run and monitor different tasks of the workflow—as well as all objects going in and out of the tasks.

**Figure 2.**
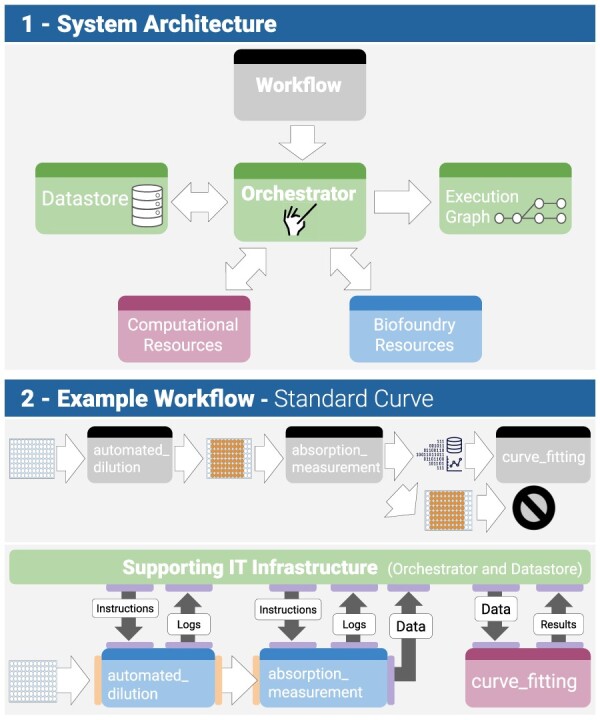
Overall system architecture and example workflow. The same color scheme was used as in Figure 1.

The system architecture is shown in Figure 2. The workflow is encoded in a DAG (called a model graph), which, in turn, instructs the workflow module to undertake a sequence of operations. The execution of the workflow is coordinated by the orchestrator (in our case Airflow (22)), which interacts with all the elements of the workflow system as follows:

- The orchestrator recruits and instructs the biofoundry resources (hardware and software) to undertake the workflow.
- It dispatches to the datastore (typically a vendor-neutral archive, supporting open data formats) the data from the execution of the workflow (operational data, experimental data and, also, *in silico* biodesign data), so they may be subsequently used for analysis.
- It generates an execution graph, stored in a dedicated graph database (Neo4j (23) was used), which is a log of the execution of the workflow and links all undertaken steps of the workflows to descriptions of its inputs and outputs.

**Figure 3.**
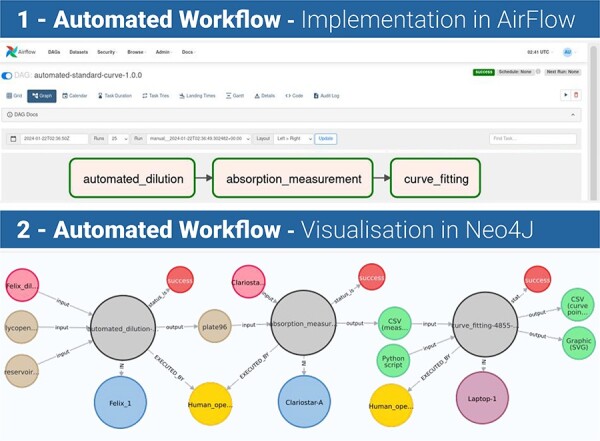
Orchestration of a simple workflow in practice.

This can be further illustrated in the simple workflow, developed for the purpose of the creation of a calibration curve (the bottom of Figure 2). The workflow comprises three steps: creation of a dilution plate on a liquid-handling platform, measurement and data analysis. The way in which the orchestrator utilizes the available resources (hardware and computational)—and the corresponding physical data flows between the orchestrator, the datastore and the available resources—is detailed in the same figure. Figure 3 introduces the software stack of our proposed solution—for the same workflow example (referred to as the ‘Automated dilution workflow’). Figure 3, section 1 shows a screenshot of the graph view in AirFlow, while Figure 3, section 2 shows a screenshot of the Neo4J Browser with the execution graph. The solution is currently being trialed at the London Biofoundry at SynbiCITE (24).

The authors believe that the power and flexibility offered by the orchestrator (at the heart of the proposed architecture) make it stand out from a number of current infrastructures (25), which are mainly concerned with the logging, storage and subsequent analysis of all experimental and procedural data. In particular, the approach enables the building of automated pipelines distributed across more than one biofoundry. In this case, while the automated platforms may be in different locations, each biofoundry will be connected to the system architecture. Instructions flow from the central infrastructure to the software layer of the platforms, to their mechanized layer and back. The orchestrator coordinates the tasks encoded into the DAG across the different biofoundries involved in the workflow.

## 4. Conclusion

The methodology described in the paper addresses automated workflow in the context of a biofoundry. As described in the paper, workflow in a biofoundry frequently results in large amounts of data, often involving higher-throughput and higher replicability. The approach described is based on a supporting IT infrastructure and the use of DAGs and orchestrators. The methodology is designed to be as generic as possible.

## Data Availability

No new data were generated or analysed in support of this research.
